# Philadelphia Medical Society

**Published:** 1841-12-11

**Authors:** 


### PROCEEDINGS OF SOCIETIES.

### PHILADELPHIA MEDICAL SOCIETY.

*Session of Nov. 27.—Dr. Reynell Coates delivered a lecture on Animal Magnetism, philosophically considered.*—The views contained in this lecture are so peculiar, and in some respects novel, as to leave us no alternative between an extended abstract and its entire omission. As the lecturer himself believes his views likely to direct the minds of other investigators to practical results of value, we have chosen the former course, although it obliges us to extend the article beyond the present number. In accomplishing the task, we have availed ourselves of the original text and notes. Whatever may be thought of the opinions expressed, some valuable facts and interesting speculations will probably repay the reader for the expenditure of his time and our space.

"*Gentlemen,*—Many of the members of this learned body are aware that the singular nervous phenomena usually included under the now unmeaning title of animal magnetism, and absurdly dignified with the title of a science by the vulgar, and even by some of the more imaginative of those investigators who may be with justice included in the brotherhood of physical or physiological philosophers, have been made a subject of extensive experiment by at least two members of the society; and that the facts obtained by these members were duly canvassed in the debate on the lecture of Dr. Pierce, delivered, if I remember correctly, in the year 1837.

These facts were principally derived from the experience of the lecturer and that of the present Professor of the Practice of Medicine in Jefferson Medical College.

Both these gentlemen very properly avoided founding any theoretical, or, rather, hypothetical opinions upon these facts, because they were insufficient to form the foundation of any rational theory erected upon acknowledged physiological data. They were declared, on all hands, inexplicable, though true. They extended, on the part of Dr. Pierce, not only to the production of the cataleptic condition in which many or all of the organs of sensation refuse the performance of their proper functions, but, also, to the causation of a condition in which the subject of experiment became *apparently* capable of recognizing the taste and properties of articles in the mouth or about the person of the operator, while perfectly incapable of perceiving the taste or properties of similar articles when placed in or upon his or her own mouth or person. Further than this the experiments were not carried. On the part of Dr. Mitchell, (J. K.,) they proceeded little beyond the production of the cataleptic state, with the destruction of the natural sense of touch or feeling, and certain other senses, to such a degree that the lesser tests usual on these occasions were applied without awakening any impressions. The most pungent substances were introduced in the nostrils, and the fumes of the strongest liquid ammonia permitted to be inhaled by the same route, by one or both of the operators, sometimes until the skin was excoriated by the stimulus, without any sign of reluctance in the patient. It is well known that teeth were drawn by Dr. Mitchell, on one occasion, with a similar result.

"There are facts enough, therefore, already in possession of this society to prove, beyond the power of cavil, the actual production of catalepsy *apparently* by the employment of certain manipulations, and, perhaps, grimaces;—facts derived from those about whose good faith no question can arise—whose interest could not be, in any manner, promoted even by their avowal, and who both were made to suffer, we believe, by the justice which they did to science in simply stating the truth—the unvarnished truth—in public. In establishing this point, then, there is no necessity to refer to other experiments of the same nature, by the same or other gentlemen, which were pushed, in some instances, to an extent against which it would not be improper to enter a solemn protest. It is equally unnecessary, at present, to quote the phenomena displayed in certain recent public exhibitions in this city, of which I heartily disapprove, but have been compelled by the duty of the professional critic and journalist to witness. But in making a public avowal of this disapproval, justice requires that I should acknowledge the courtesy and frankness with which the unprofessional operator has facilitated my investigation of those phenomena.

"I will not detain the society with any account of the amputations of Jules Cloquet, or the horribly cruel and abominable tests of insensibility which have been tried in different parts of Europe upon patients in the cataleptic condition, artificially produced, ending, it is said, in one instance, in the death of the subject upon the chair of the operator. It would be an insult to your good sense to suppose you ignorant of a sufficient amount of this kind of evidence, independently of the personal experience of your fellow members; and it would be equally insulting to doubt your capacity to estimate the value of that evidence so far as the simple facts are concerned.

"The existence of the cataleptic condition, as occasionally produced *apparently* by certain manipulations *or* by certain exercises of individual will, is no longer a legitimate subject of debate among well informed physicians. I know of no such individual who has the hardihood to express a positive disbelief of it, though there are many who smilingly or fearfully decline all expression of opinion on the subject, well knowing that an affirmative avowal would produce a loss of influence with the vulgar or the ignorant, and limit, to a certain *appreciable* extent, the *immediate emoluments of practice.* There are a few, it is true, who continue to doubt, without an absolute denial, on this one point, and for these I entertain a high respect, though firmly convinced that a few moments carefully spent in thought would remove their doubts.

"The condition to which allusion has been made, is termed, for the present, *cataleptic*, because, although the term catalepsy infers the existence of muscular spasm, and muscular spasm is only occasionally present in what is now usually termed the Mesmeric State, yet there is no ordinary disease which approaches so nearly to this condition in its rational signs, if we except certain stages in the progress of chronic hysteria. It is unnecessary to describe, before so enlightened an audience, the peculiar appearances presented by cases in which the various proceedings of what are mis-termed 'the animal magnetisers' have been successfully adopted, whether those proceedings have been productive of clonic and tonic spasm, or of an insensible condition accompanied by unusual relaxation of the muscles; for both these forms are witnessed in the artificial production of the disease, according to the temperament or idiosyncracy of the subject. But—waiving, for the present only, the supposed transfer of the senses which the experiments of Dr. Pierce would lead us to infer, I do positively aver that there is nothing in this condition which is either unusual or peculiar. The only anomaly or originality consists in the peculiar manner in which the condition is supposed to be produced.

"No man, who is at all familiar with cases of chronic hysterical convulsions, as often exhibited in the wards of large hospitals, can have failed to notice patients in whom—either with or without the existence of spasm, or with these two states of the muscular system regularly alternating with each other,—all power of voluntary motion and consciousness have vanished for hours together. I have made it a special subject of inquiry, in such cases, to determine how far there existed an incapacity to receive the ordinary impressions of the senses. I have subjected patients in the Pennsylvania Hospital, prior to the year 1823, to most of the admissible tests of the animal magnetisers, (so styled,) such as plunging pins in the flesh to the depth of nearly an inch, making the patient inhale by the nose, for a short time, the fumes of aqua ammonia, subjecting the eye to the direct rays of a strong light or the sudden approach of [foreign substances,—*and in some individuals, at certain periods*, these tests awakened no consciousnesss whatever. In two other instances, occurring in and near Meadville, Pennsylvania, in 1829, or 1830, precisely the same condition was produced by the exercises of a camp-meeting in the neighbourhood. One of these patients remained for more than two days absolutely insensible to ordinary impressions, except, as it seemed, through the ear. She appeared to hear all that passed; for, in the midst of a general rigidity of muscles which rendered her body and extremities perfectly motionless, she talked incoherently and wildly *of more worlds than one or two*, and mingled with her discourse impertinent replies to remarks made by her friends and others in the room. Some of her observations appeared to refer to the acts and attitudes of visitors, and would have been supposed by any believer in clairvoyance to furnish evidence of the existence of that singular power, but I discovered nothing of this kind which might not have been accounted for upon the suppositon of an intense acuteness of hearing. She was twice bled, cupped upon the temples, and again on the back of the neck, and mustard plasters were applied to the inner side of the leg. The latter were allowed to remain, contrary to my express caution, for several hours; and one of them gave rise to a very troublesome ulceration, requiring to be treated for several weeks. Yet at no time was there exhibited the slightest symptom of consciousness. Both the patients just mentioned were repeatedly pinched on the arms and neck, sometimes until the part was blue. They were pricked with pins, and hairs were drawn out by the roots without producing any sensation whatever. I have no doubt a limb might have been amputated without awakening sensation in either of them.

Waiving the alleged sympathy between the subject and the magnetiser, there is an absolute identity between this hysterical catalepsy or the catalepsy from pseudo-religious enthusiasm, and that resulting from mesmeric manipulation.

"Though there is nothing more wonderful in the degree of insensibily observed in such cases than in ordinary palsy, yet it is made a matter of wonder by the ignorant and the careless thinker, who sees nothing remarkable in palsy, merely because the latter disease is presented, almost daily, in the public streets; while the hysteric catalepsy is met with only occasionally, and in the wards of large hospitals; and the mesmeric catalepsy is produced under circumstances often open to the suspicion of deception.

"There are many persons who still continue to regard a *seeming sleep*, attended with a loss of feeling so complete that amputation will not even wake the patient, as something anomalous in nature. To disprove this, I will narrate two cases illustrating the disorder of the nervous system, resulting from an over-dose of alcohol."

Here the lecturer quoted the history of two cases occurring within three or four years of the present time, in the practice of the Pennsylvania Hospital.

In the first of these, the patient was wandering along the road in the low ground below Philadelphia, in a state of intoxication. He paused by the side of a ditch designed to drain the meadows, to wash his feet, which were muddy with travel. He fell asleep with both feet and one hand in the water, and his seat was upon the wet margin of the ditch. The night became—unexpectedly—intensely cold, and the parts submerged were surrounded by solid ice. He was found in the morning, unable from debility to extricate himself. The ice was broken, and he was conveyed to the Hospital with sphacelus of the three extremities and the scrotum, and died there.

In the second case, a man deeply intoxicated wandered into a tavern, and being unable to leave it, was placed in a chair near a stove *red hot with the combustion of anthracite*. During the night, he slid down upon his seat until his knee came in contact with the heated iron, and was found in that attitude in the morning, by the family, *still asleep!* He was conveyed to the Hospital, and placed under the charge of the surgeons of that house. The patella was burned to charcoal. The burn extended to more than one-third of the diameter of the head of the tibia and the condyles of the femur, laying open the knee joint to its centre. The condition and habits of the patient precluded the operation of amputation; he lived many days, and died of exhaustion. Both these cases are fresh in the memory of many members of the Society.

The lecturer then recurred to the acknowledged fact that the mesmeric or "magnetic" manipulations really do disturb the equilibrium of the nervous system, and that, in character, the disturbance resembles hysteria, or catalepsy. He stated that the habit of attributing the effect to the action of the *imagination*, did not in any degree vitiate the fact. Imagination often determines life and death. Its action on the body is a legitimate subject of physiological research, as are all the passions. He alluded to the sudden death, from joy, of the old door-keeper of the first Congress, on the announcement of the victory of the allied armies over Lord Cornwallis. He pointed out the folly of those who, on finding that all the effects attributed by Perkins to his metallic tractors, could be produced by the same manipulations without the aid of tractors, condemned the obvious facts as humbug, together with the unfounded doctrines based upon those facts.

"The simple fact of the nervous disturbance following certain manipulations, is enough to prove either that the mind of one individual can control the mind of another, and thus react upon the nerves; or, that one nervous system can act directly upon another, at sensible distances.

"Now, it is of the utmost importance to the rational understanding of many really curious phenomena, that we should determine at once whether the condition called mesmeric is essentially the result of psycological or physical causes; because the idea of its purely mental origin is the source of all the wild, the absurd, the monstrous, the infidel, the sacrilegious opinions which have been grafted upon the miscalled theory of mesmerism, by the folly of those who are incapable of rigid physiological deduction. If we can prove that the operation is not effective through the influence of immaterial thought, we clear the subject at once of all the jargon of the pseudo-metaphysical animal magnetizers. It is only with those inconsequent reasoners who regard the mental powers as mere functions of the nervous system,—a sect with whom I have been waging war even to the knife, for the last three years,—that we shall hear the mind mentioned as other than very indirectly concerned in the history of mesmeric hysteria or catalepsy.

"Fortunately it is in my power, without reference to a thousand well-authenticated facts already on record,—the true bearing of which has not been perceived,—to prove conclusively, from personal observation, that the controlling or modification of the mind has no necessary connection with the production of the cataleptic condition."

Here the lecturer introduced the two observations narrated in his article published in our forty-fifth number for the present year, (page 716.) In both these cases, all the spasmodic phenomena were produced while the mind remained intact.*

"With this expose of local mesmeric action, all the silly dogmas of transfusion of the will and separation of the soul from the body, which disgrace the *doctrine* of certain dreamers, crumble into dust, and the subject is reduced within its proper limits as a subject of plain physiological research into physical actions.

"Two things are fully proved, however, by these facts. One of them is of modern demonstration. The other never was denied in any age.

"1st. The mind of any individual, by its reaction on the nervous system, may modify its functions, and—to borrow a phrase from chemistry—using it allegorically—produce a change in its affinities.

"2d. The nervous system of one individual *may* exert a physical influence over that of another, in such a manner as most seriously to modify its functions, without the intervention of the anterior portion of the brain of the subject, and without there being any legitimate reason for supposing that any portion whatever of the brain participates in the impression.

"It follows from these predicates, that, so far as the individual subjected *to the experiment* is concerned, the apparent mental phenomena observed in Mesmeric catalepsy and hysteria, are not causative of the condition, but consecutive upon it. They may be present or absent, and may vary in degree and character—as the mere muscular affection is known to do—with all the peculiarities of temperament and idiosyncracy. This, at once, explains why some individuals are more powerful than others, as operators, and why some subjects are more liable than others to the influence of Mesmerism.

"It is a popular, though altogether erroneous impression, that susceptibility to this species of morbid action is a proof of relative mental inferiority in the subject. Observation has not held out this position, nor could it be deduced legitimately from any rational attempt to explain the phenomena of Mesmerism."

The lecturer proceeded to assure the society that most of the subjects in whom he had witnessed this cataleptic or hysterical condition, were persons of superior mental endowments, in one instance very superior to those of the operator. The debility or morbid excitability was only discoverable on ordinary occasions, in the portions of the nervous system exterior to the brain. *The cause* being obviously a physical influence of one nervous system over another, he contended that *the effect* must inevitably be mutual between the subject and the operator—by the law of the equality of action and reaction,—and that the difference in apparent effect could only be the result of the accidental difference of susceptibility in the whole or some one portion of the nervous systems of the two parties in an experiment. He stated that the flexor muscles of his own forearm had been subjected to spasm on the first occasion of

* We have since learned that the literary gentleman who was the subject of the first of these observations, has become, by habit, more susceptible of the action of the causes of this nervous disturbance, so that he displays even the motions performed at the will of the operator, which have been rendered so familiar to the public by recent exhibitions; yet it has been found impossible to affect his mind in the least. He states that these motions are beyond his control, though recognized by his consciousness, and that they are performed without volition on his part. R. C.

his being placed in communication with a patient in the mesmeric catalepsy, and that on all succeeding occasions they had been affected with pain. This phenomenon he would have attributed to accident, had not the hand of his friend Dr. Caspar Morris been rendered somewhat paralytic for many minutes under the same circumstances. This mutual influence of one nervous system over another he now believed to be universal, though generally too slight to be recognised.

Dr. Coates spoke of the importance of studying this influence—if true—and believed that we are on the eve of the discovery of a new general law in Physiology, though but a few bare facts in relation to this subject have been, as yet, substantiated. He thought that many questions now considered as within the domain of Moral Philosophy and Psycology, might be illuminated by the prosecution of truly philosophical investigations into the history of this mutual nervous influence, to which it is at present an utter folly to attach the names of electricity, magnetism, galvanism, or gravitation, there being at present not the shadow of a reason for such connexion. Among the curious subjects which might possibly admit of a physical explanation by such means, he instanced the aptitude of minds in society to assume a common train of thought and feeling—grave or gay—as if by contagion; the singular approximation in appearance between dissimilar individuals after long continued matrimonial connexion; and the physico-moral peculiarities of races in man and animals, as well as some of those families, parties, and religious sects. The observations on this branch of the subject were curious and novel, but our space and the objects of our Journal permit us only to indicate them.

The length and variety of this lecture compels us to defer the very peculiar views of the speaker on the seemingly mysterious subject of *clairvoyance.* At present we will only remark that he considers it as a distinct sense existing in all animals, not identical with vision, and not developed by disease in man beyond its natural condition in some inferior animals: and, that it is found,—though only *typically*, and scarcely recognizable—in the healthy man.

(*To be continued.*)

